# *Lacticaseibacillus paracasei* LC86 mitigates age-related muscle wasting and cognitive impairment in SAMP8 mice through gut microbiota modulation and the regulation of serum inflammatory factors

**DOI:** 10.3389/fnut.2024.1390433

**Published:** 2024-05-30

**Authors:** Yihui Cai, Yao Dong, Mei Han, Manfei Jin, Huan Liu, Zhonghui Gai, Kang Zou

**Affiliations:** ^1^Germline Stem Cells and Microenvironment Lab, College of Animal Science and Technology, Nanjing Agricultural University, Nanjing, China; ^2^Stem Cell Research and Translation Center, Nanjing Agricultural University, Nanjing, China; ^3^Department of Food Quality and Safety, Shanghai Business School, Shanghai, China; ^4^Department of Animal Experiment, Chengxi Biotech, Shanghai, China; ^5^Department of Research and Development, Wecare Probiotics Co., Ltd., Suzhou, China

**Keywords:** muscle wasting, cognitive impairment, *Lacticaseibacillus paracasei* LC86, gut microbiota, inflammatory factors regulation

## Abstract

**Purpose:**

Chronic inflammation contributes to the decline in muscle strength and cognitive abilities associated with aging. This study aims to clarify the effects of oral administration of *Lacticaseibacillus paracasei* LC86 on these age-related declines, as well as its impact on the composition of gut microbiota.

**Methods:**

Senescence-accelerated mouse prone 8 (SAMP8) mice received a 12 week regimen of LC86 (1 × 10^9^ CFU/day). Muscle strength was assessed through forelimb grip strength and four-limb hanging tests. Cognitive function was evaluated through behavioral performance tests, and changes in gut microbiota were analyzed.

**Results:**

Administration of LC86 significantly enhanced muscle strength, demonstrated by increased grip strength and higher glycogen content in the gastrocnemius muscle (*p* = 0.041, *p* = 0.017, and *p* = 0.000, respectively). Behavioral tests suggested that LC86 mitigated age-related cognitive decline. Furthermore, there was a significant decrease in serum pro-inflammatory cytokines, such as IL-6, TNF-α, and MCP-1 (*p* = 0.002, *p* = 0.000, and *p* = 0.005, respectively), and an elevation in the anti-inflammatory cytokine IL-10 level (*p* = 0.000). An increase in hepatic antioxidant capacity was observed. Significant changes in the gut microbiota composition were noted, including increased populations of *Bifidobacterium* and *Lactobacillus* and decreased levels of *Escherichia*/*Shigella* and *Bacteroides*.

**Conclusion:**

The findings suggest that LC86 supplementation mitigates muscle weakness and cognitive impairment in aging SAMP8 mice, potentially through the modulation of inflammation and gut microbiota composition. LC86 emerges as a promising candidate for ameliorating the decline of muscular and cognitive functions associated with aging.

## Introduction

As the global demographic shifts towards an older population, with individuals aged 60 years and above becoming increasingly prevalent, the challenges of aging, including organ function decline and associated muscle wasting and cognitive deficits, are of growing concern (1, 2). These age-related conditions, including sarcopenia and cognitive decline, not only diminish quality of life but also heighten the risk of falls and fractures, with significant implications for public health (3, 4).

Aging process is often marked by the elevated levels of pro-inflammatory cytokines, such as tumor necrosis factor α (TNF-α) and interleukin (IL)-6 (5), which could potentially impede muscle protein synthesis, accelerate muscle protein degradation, and disrupt neurotransmitter release and neuronal function, ultimately contributing to muscle atrophy and cognitive dysfunction (5, 6). Moreover, the high metabolic demands of muscle and brain tissues render them particularly vulnerable to oxidative damage from free radicals, which accumulate with age and can exacerbate muscle and cognitive impairments (7, 8).

Emerging evidence underscores the critical role of the gut microbiota in maintaining human health, with dysbiosis contributing to compromised intestinal barrier integrity, release of noxious substances, and adverse outcomes for muscle and neural tissues (9). The gut microbiota is also implicated in modulating metabolites, immune responses, and neurotransmitter dynamics, further influencing muscle and cognitive functions (10). Consequently, age-associated muscle wasting and cognitive impairment are increasingly linked with inflammatory profiles, oxidative stress, and gut microbiota composition, spurring research to unravel these complex interactions and develop effective preventive and therapeutic interventions (11).

Probiotics are recognized for their capacity to enhance gut microbiota by suppressing pathogenic bacteria and fostering beneficial microbial communities, thereby preserving intestinal barrier function and mitigating inflammation. Through the suppression of inflammatory cell activation and signaling pathways, probiotics can lower concentrations of pro-inflammatory cytokines (12–14). They also bolster host antioxidant defenses by elevating levels of enzymes like superoxide dismutase (SOD) and glutathione peroxidase, thereby attenuating oxidative stress and free-radical-induced damage (15). Furthermore, probiotics can enhance gut barrier function and mitigate oxidative damage by modulating gut-derived metabolites, such as short-chain fatty acids (16). For instance, *Lactobacillus plantarum* TWK100 has been reported to improve muscle mass and strength, and reduce muscle atrophy in aged rats through the reduction of intestinal inflammation and enhancement of antioxidant capacity (17). Similarly, supplementation with *Lactobacillus paracasei* PS23 has been shown to mitigate age-related cognitive decline in SAMP8 mice by enhancing antioxidative defenses and modulating inflammatory markers, suggesting potential therapeutic benefits against aging effects (18). Additionally, intervention with *Lactobacillus paracasei* GKS6 in SAMP8 mice has significantly delayed aging, enhanced muscle strength and fiber count, and improved liver antioxidant activities, reducing oxidative stress markers. These probiotic strains act as potent antioxidants, potentially mitigating aging-related muscle decline (19). Clinical studies have also indicated that probiotic combinations can significantly enhance cognitive function and psychological well-being, with noted reductions in serum levels of inflammatory markers, particularly IL-6 and high-sensitivity C-reactive protein, alongside cognitive and mood improvements (20). Additionally, consumption of *L. rhamnosus* GG has been associated with increased muscle mass, reduced muscle damage, and decreased production of inflammatory markers in the elderly (21). Therefore, the discovery and characterization of new probiotic strains capable of efficiently improving muscle strength and cognitive function, and the elucidation of their mechanisms, are pivotal for guiding the development of probiotics for public health applications.

The present study utilizes the senescence-accelerated mouse prone 8 (SAMP8) model, which exhibits pronounced symptoms of premature aging such as alopecia and reduced lifespan, to assess the potential of *Lacticaseibacillus paracasei* LC86 against age-related muscular and cognitive decline (22). While previous studies have highlighted the anti-aging potential of probiotics, particularly regarding antioxidative functions and muscle strength, investigations into gut microbiota alterations in aging models remain limited (23–25). LC86, within a probiotic consortium, has shown to significantly bolster intestinal stem cell activity, enhance intestinal barrier integrity, and modulate oxidative and inflammatory responses. Moreover, LC86 contributes to a beneficial restructuring of the gut microbiome, enhancing populations of short-chain fatty acid producers and upregulating anti-inflammatory and antiradiation metabolites (26). This study offers a detailed comparative analysis between control and LC86-supplemented SAMP8 mice, aiming to provide a comprehensive understanding of the mechanisms of LC86 and affirm its therapeutic promise in promoting health span and longevity.

## Materials and methods

### Strain culture and preparation

*Lacticaseibacillus paracasei* LC86 (deposit number: CGMCC No. 1.12731) was obtained from Wecare Probiotics Co., Ltd., Suzhou, China. This strain was cultured in De Man, Rogosa, and Sharp (MRS) broth, maintaining the culture at 37°C for a period of 16 h to ensure optimal growth and viability. Post-incubation, the bacterial cells were harvested by centrifugation at 6000 × g for 8 min at 4°C. The resultant pellets were resuspended in sterile water to achieve a concentration of 5 × 10^9^ CFU/mL and stored at 4°C until usage. This bacterial suspension was freshly prepared weekly during the experiment and administered to the mice in a dose of 1 × 10^9^ CFU/mL (27).

### Grouping of mice and the intervention

**Figure 1:**
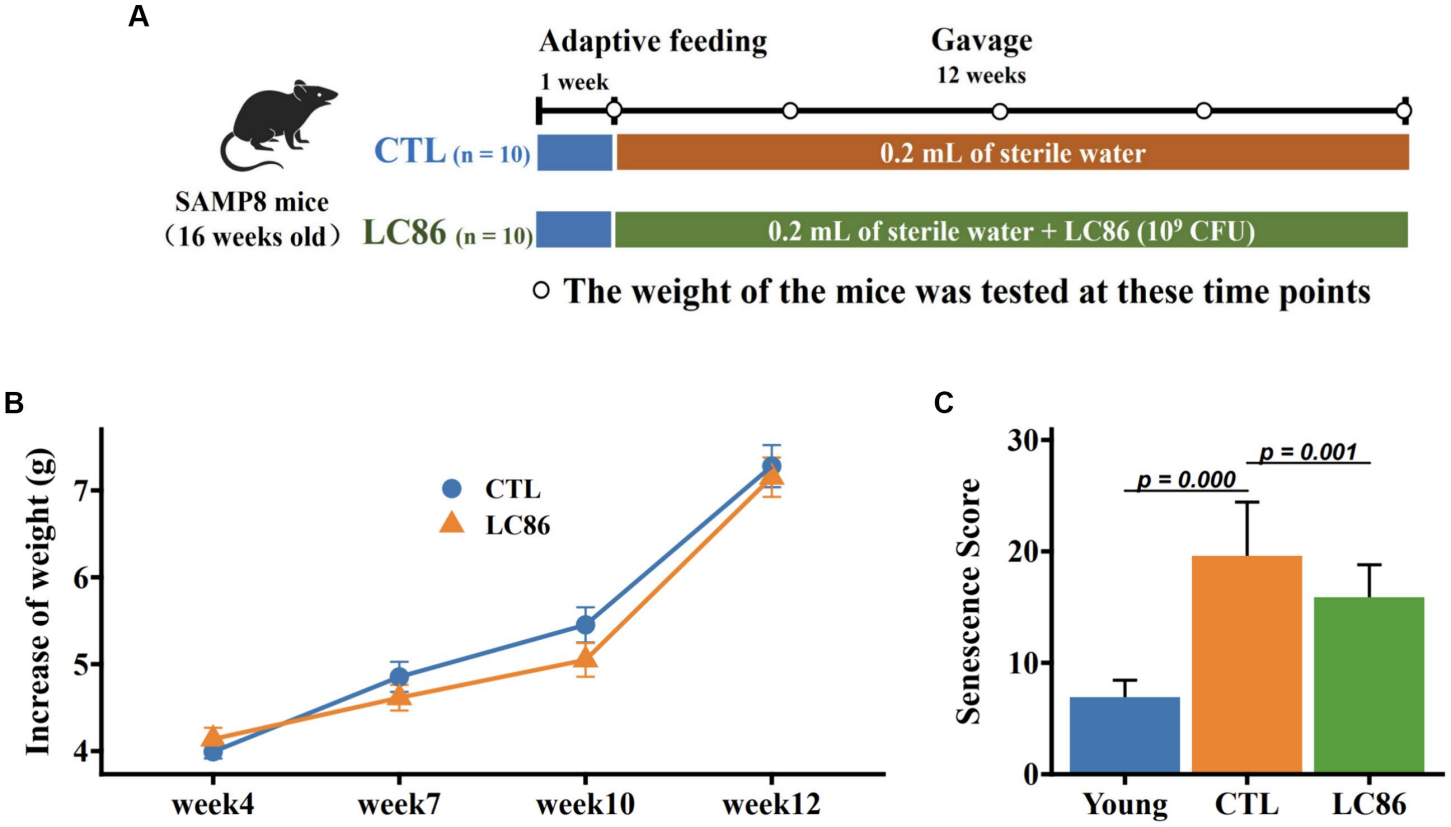
Experimental design, change in body weight of SAMP8 mice, and senescence score. The experimental intervention period was 12 weeks using *Lacticaseibacillus paracasei* LC86 **(A)**; body weight was measured from week 1 to week 12 **(B)**; senescence score **(C)** was measured before and at the end of the experiment.

Twenty 16 week-old specific pathogen-free male SAMP8 mice were obtained from Shanghai Laboratory Animal Center, and this study was approved by the Animal Care and Use Committee of Shanghai Laboratory Animal Center (No: 2023033011). Mice were maintained in an environment with a constant humidity (65% ± 5%) and temperature (25 ± 2°C) and a 12 h light/dark cycle. A suspension of the LC86 strain was prepared as previously described for daily administration to mice in the intervention group (27). Building on prior research that demonstrated the beneficial effects of probiotics on the SAMP8 model (18), the 20 mice were equally divided into two groups using a simple randomization method. The control group (CTL), serving as the model control with 10 SAMP8 mice, received 0.2 mL of sterile water as a vehicle treatment daily. Parallelly, the intervention group, which also consisted of 10 SAMP8 mice and received treatment with *L. paracasei* LC86 (LC86), was administered a daily volume of 0.2 mL sterile water containing 1 × 10^9^ CFU of LC86. This administration occurred over a period of 12 weeks. Figure 1A illustrated the experimental procedure, which includes a 12 week intervention period accompanied by regular assessments of mouse body weight and systematic collection of fecal samples. Fecal samples were collected at the outset of each experimental week, specifically between 8 and 9 AM, with approximately 2.0 g of feces obtained from each mouse. These samples were immediately placed into sterile 2 mL centrifuge tubes, snap-frozen in liquid nitrogen, and then transported to the laboratory for storage at −80°C until further analysis. At the end of the experiment, the mice were anesthetized using isoflurane at 2% and euthanized by CO_2_ asphyxiation to minimize any potential discomfort or distress. Orbital blood samples were collected and centrifuged at 4,500 × g for 15 min to obtain serum samples, which were then stored at −80°C until analysis. Liver samples were collected and fixed in a 4% formaldehyde solution until histological analysis. Brain and colon samples were collected immediately and stored at −80°C.

### Assessment of senescence

The degree of aging in SAMP8 mice was assessed using a graded scoring system, which assessed mouse behavior and appearance as the indicators of aging. The scoring criteria are provided in Supplementary Table S1. Each indicator was represented by five levels of severity and scored by the investigator, and scores for each indicator were combined to obtain a total score for the degree of aging for each mouse.

### Testing of muscle strength

The muscle strength of the mice was determined using the forelimb grip strength and four-limb hanging tests. In the forelimb grip strength test, the mice gripped a horizontal bar with their forelimbs while the use of their hindlimbs was prohibited by tethering them to the tail. The maximum grip force was recorded over 10 trials, and grip force values were expressed as grip force (gm f). In the four-limb hanging test, mice were placed hanging upside down in wire cages, with all of their limbs gripping the wire, and then allowed to hang for a few seconds. The time that the mice were able to remain suspended was recorded. The test was repeated three times and the time was averaged for each mouse. The results were expressed as the holding impulse (N sec).

### Analysis of muscle glycogen content

The glycogen content of mouse gastrocnemius muscle was determined as follows: 100 mg of muscle tissue was homogenized in 0.5 mL of cold perchloric acid and then centrifuged at 15,000 × g and 4°C for 15 min, after which the supernatant was collected. Muscle glycogen content (expressed as mg/g muscle) was determined using a muscle glycogen assay kit (Nanjing Jiancheng Bioengineering Institute, Nanjing, China).

### Open field test

Mice were carefully placed in a Plexiglas activity chamber (25 × 25 × 40 cm) and were allowed to freely explore the chamber for 10 min. The behavior of the mice in this chamber, including their motor activity and time spent in the central area, was observed and recorded. After each experiment, the chamber was cleaned with 70% ethanol to neutralize the smell of urine and feces and reduce olfactory interference in the next experiment.

### Morris water maze test

The spatial learning and memory of the mice were tested using the Morris water maze (MWM) test. The test was performed in a circular pool with a diameter of 100 cm, a height of 40 cm, and a water depth of 30 cm, and the water temperature was maintained at 26 ± 1°C. The mice were trained to find the platform twice daily for 5 days. If the mice were unable to reach the platform within 60 s, they were led to the platform. On day 6, the platform was removed and the mouse’s swimming path was tracked with AnimalTracker to record the time it took the mouse to reach the location where the platform had been.

### Determination of brain neurotransmitter concentrations

Samples of the hippocampus and bilateral striatum from the mouse brains were homogenized and centrifuged at 12,000 × g and 4°C for 10 min. The supernatant was filtered through a 0.22 μm-pore membrane, and the filtrate was used for high-performance liquid chromatography–mass spectrometry (HPLC-MS) detection. A Thermo Vanquish Ultra Performance Liquid Chromatography System from Thermo Fisher Scientific (Waltham, MA, United States) and an ACQUITY UPLC HSST3 column from Waters (Milford, MA, United States; 2.1 × 150 mm, 1.8 μm) were used for HPLC. The flow rate was set to 0.25 mL/min, the column temperature to 40°C, and the injection volume to 2 μL. For MS, a Thermo Q Exactive Focus mass spectrometry detector from Thermo Fisher Scientific was used, and data acquisition was performed with an electrospray ionization source in positive and negative modes.

### Determination of serum inflammatory factors

Serum concentrations of the proinflammatory factors TNF-α, IL-6, and monocyte chemoattractant protein-1 (MCP1) and the anti-inflammatory factor IL-10 were measured using enzyme-linked immunosorbent assay (ELISA) kits. All measurements were performed according to the manufacturer’s instructions (Jiangsu Meimian industrial Co., Ltd. Yancheng).

### Hepatic antioxidant capacity assay

To evaluate the hepatic antioxidant capacity in mice, 25 mg of liver tissue samples from each mouse were uniformly suspended in 250 μL of radioimmunoprecipitation assay buffer and centrifuged at 1,600 × g and 4°C for 10 min to collect the supernatant. The concentrations of SOD and catalase (CAT) in the liver tissues and the concentrations of glutathione (GSH) and malondialdehyde (MDA) in serum samples were determined using ELISA kits according to the manufacturer’s instructions (Jiangsu Meimian industrial Co., Ltd. Yancheng).

The method employed for staining liver tissues is based on protocols outlined in prior studies (28, 29). Specifically, Liver tissues were embedded in paraffin, sectioned at 4 μm thickness, and incubated in the dark at 37°C for 30 min. Subsequent to incubation, the sections were exposed to 5 μmol/L dihydroethidium to elicit ethidium fluorescence. The intensity of this fluorescence was then assessed as a measure of hepatic reactive oxygen species (ROS) content.

### *16S rRNA* gene sequencing and data analysis

The *16S rRNA* gene analysis procedures were as described in previous studies (27, 30, 31). Briefly, the V3–V4 region of the *16S rRNA* gene was amplified with specific primers (341F and 806R). Double-end sequencing (2 × 300 bp) of the amplicons was performed using a MiSeq platform (Illumina, San Diego, CA, United States). The raw sequence data were filtered through the lowest quality USEARCH software to obtain amplicon sequence variants (32). Alpha diversity was assessed using the vegan package. Beta diversity was assessed using principal coordinate analysis (PCoA) and permutational analysis of variance (PERMANOVA) significance tests performed using the adonis 2 function of the vegan package (v2.6-4) (33). Linear discriminant analysis effect size (LEfSe) analysis was performed online using the microeco package (34).

### Statistical analysis

Data are depicted as the mean ± standard deviation (SD), offering an indication of variability and precision within the measured outcomes. The unpaired Student’s *t* test and Mann–Whitney U test were used for the analyses of parametric and nonparametric data, respectively. Plots were generated using the ggplot2 package (35), providing a robust graphical representation of the findings. Correlation analysis was performed using the psych package, and heatmaps were drawn using the pheatmap package. To discern the alpha diversity within microbial communities, we compared both the richness, as indicated by the Chao1 index, and the diversity, as gauged by the Shannon index, across experimental cohorts. We explored the beta diversity through principal coordinate analysis (PCoA), which enabled us to elucidate structural variations in microbial communities between the groups under study. The identification of distinct microbial biomarkers for each group was performed using linear discriminant analysis effect size (LEfSe) based on abundance metrics (36). Differential analyses of gut microbiomes were conducted with the Statistical Analysis of Metagenomic Profiles (STAMP v2.1.3) package, offering a robust platform for comparing complex metagenomic data^1^. We also undertook Pearson correlation analyses to interrogate the associations between gut microbiota compositions and serum parameters in our SAMP8 model. All statistical analyses were performed using R software (version 4.3, https://www.r-project.org/), with a predefined alpha threshold of *p* < 0.05, adhering to the standard conventions for statistical significance (37).

## Results

### Impact of LC86 on body mass and senescence scores in SAMP8 mice

The SAMP8 mice serves as an animal model for aging research, exhibiting premature senescence from 16 weeks of age. Body mass evolution throughout the study showed SAMP8 mice gained an average of 6–7 grams, with no marked difference between the LC86-treated and control (CTL) cohorts (Figure 1B). Aging severity was evaluated for each mouse at study conclusion, with the CTL group SAMP8 mice displaying pronounced senescence traits. By contrast, the LC86 cohort exhibited significantly attenuated senescence scores (Figure 1C, *p* = 0.001), suggesting that LC86 administration may decelerate aging in SAMP8 mice.

### Influence of LC86 on muscular strength and glycogen accumulation in SAMP8 mice

**Figure 2:**
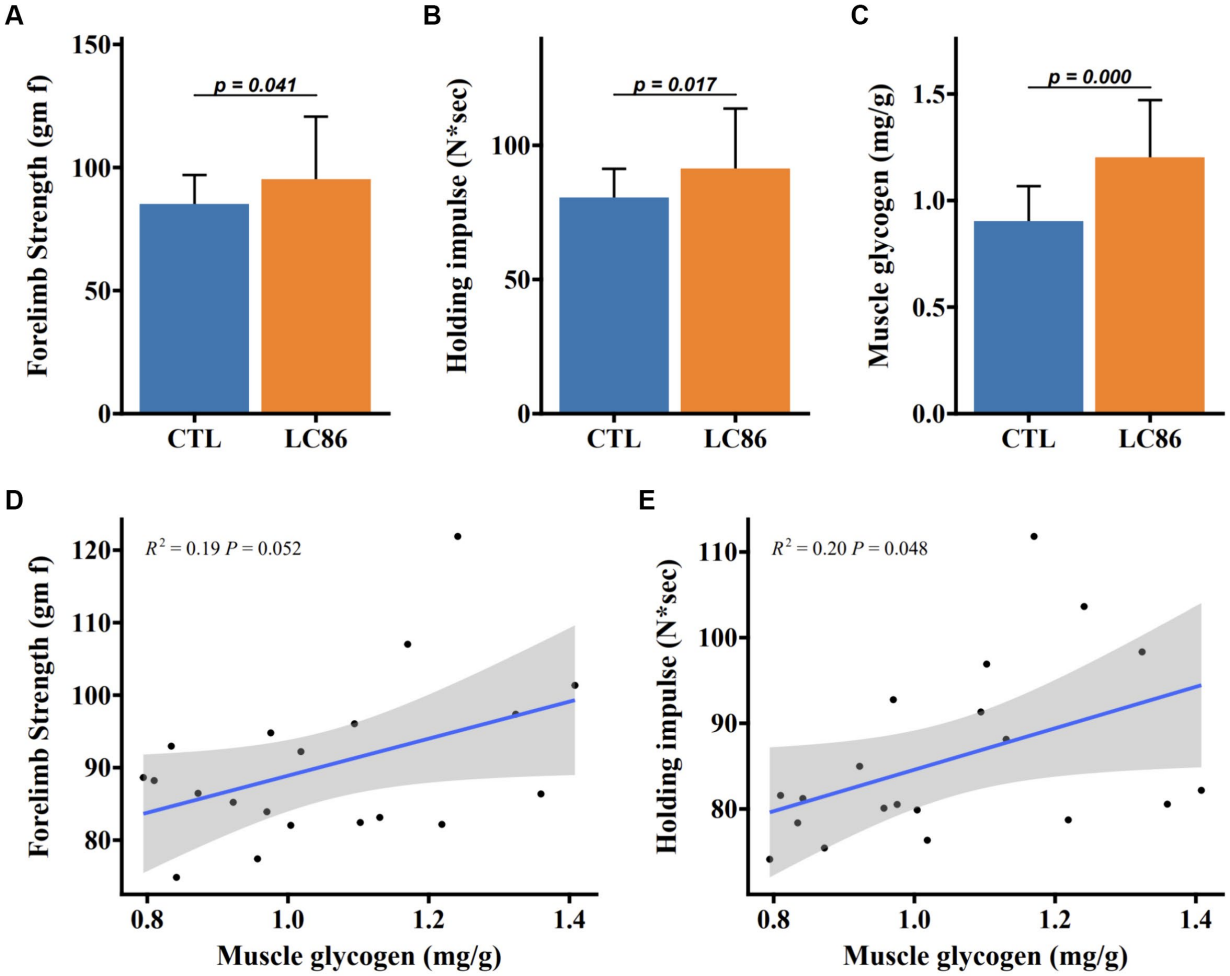
*Lacticaseibacillus paracasei* LC86 improved muscle strength in SAMP8 mice. Effect of LC86 on forelimb grip strength **(A)**, limb catching ability **(B)**, and muscle glycogen content **(C)** in gastrocnemius muscle of SAMP8 mice. The correlation between the glycogen level in the gastrocnemius muscle and the forelimb grasping force **(D)** and the ability to catch the limbs **(E)** was analyzed in the mice.

Muscular function in SAMP8 mice was assessed through forelimb grip strength and endurance-based hanging tests. Mice receiving LC86 demonstrated enhanced forelimb grip strength relative to CTL counterparts (Figure 2A). Moreover, LC86 treatment was associated with a marked reduction in age-correlated holding fatigue (Figure 2B) and a significant elevation in muscular glycogen levels (*p* < 0.05, Figure 2C). Positive correlations were observed between muscle glycogen reserves and both forelimb grip strength (*p* = 0.052, Figure 2D) and limb retraction proficiency (*p* = 0.048, Figure 2E), indicating that LC86 may mitigate age-related muscular strength decline in SAMP8 mice by promoting glycogen storage.

### Effects of LC86 on locomotor activity and neural transmitter levels in SAMP8 mice

**Figure 3:**
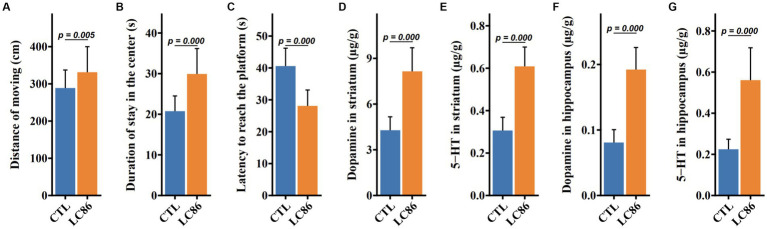
*Lacticaseibacillus paracasei* LC86 improved the cognitive performance of SAMP8 mice. In open-field experiments, LC86 improved the mice’s locomotor distance **(A)** and dwell time in the central region **(B)**. LC86 decreased the delay in reaching the platform in the water maze experiment in mice **(C)**. LC86 significantly increased dopamine **(D)** and serotonin **(E)** levels in mouse hippocampus and dopamine **(F)** and serotonin **(G)** levels in striatum.

Locomotor behavior was quantified using an open field assay, where LC86-treated mice displayed increased travel distance (*p* = 0.005, Figure 3A) and prolonged central zone occupancy (*p* = 0.000, Figure 3B) compared to CTL mice. During the Morris water maze (MWM) task (*p* = 0.000, Figure 3C), LC86-treated mice exhibited reduced latency in locating the platform, suggesting enhanced cognitive function. Neurotransmitter analysis in the hippocampus and bilateral striatum revealed elevated dopamine (DA) and serotonin (5-HT) levels in LC86-treated mice compared to CTLs (both *p* = 0.000, Figures 3D,E). These findings were paralleled in the bilateral striatum DA and 5-HT concentrations (both *p* = 0.000, Figures 3F,G), highlighting the potential neuroprotective effects of LC86 in SAMP8 mice.

### Modulation of serum inflammatory markers by LC86 in SAMP8 mice

**Figure 4:**
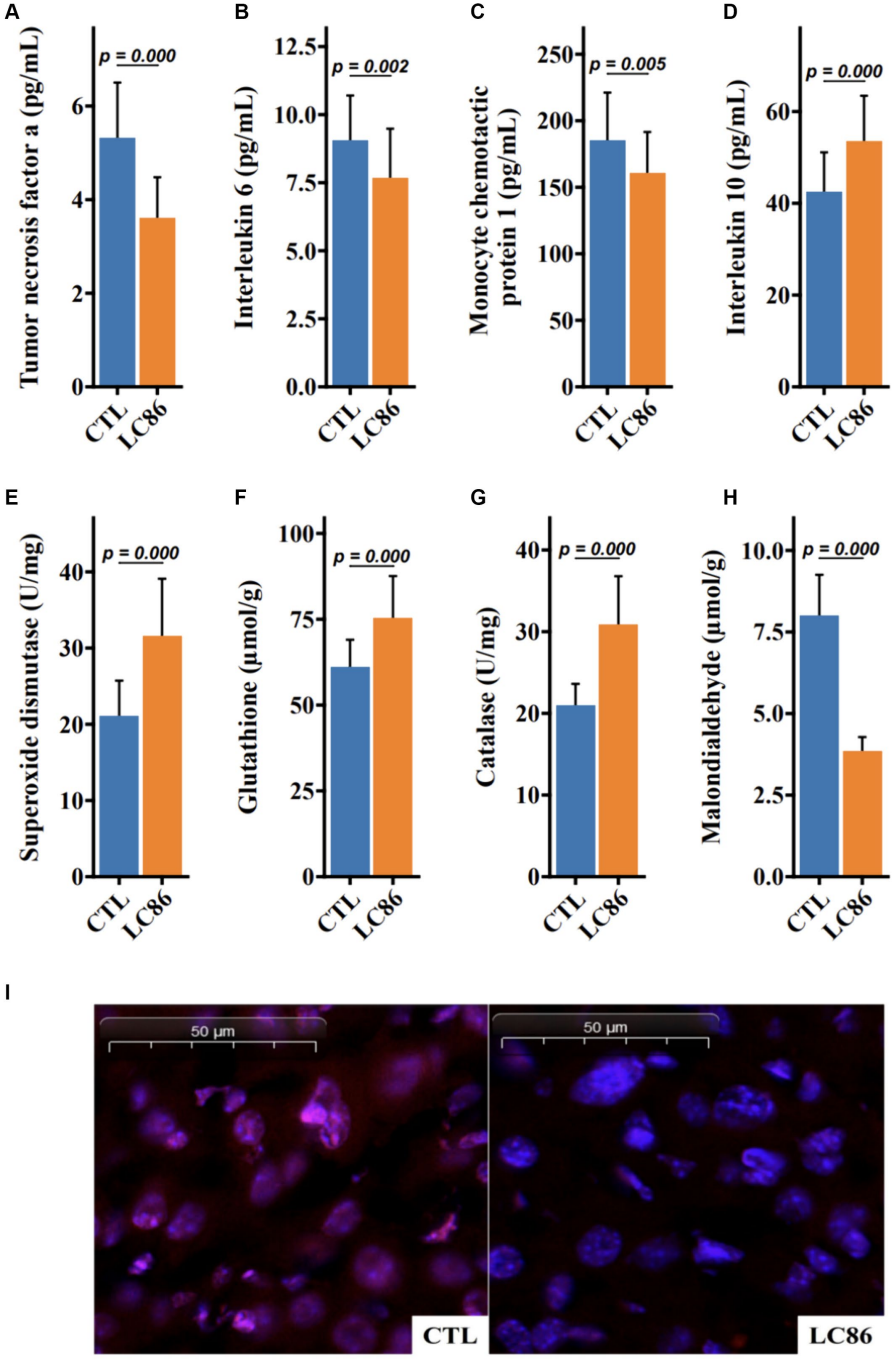
*Lacticaseibacillus paracasei* LC86 improved serum inflammatory response and increased liver antioxidant capacity in SAMP8 mice. Effect of LC86 on serum tumor necrosis factor α **(A)**, interleukin 6 **(B)**, monocyte chemoattractant protein-1 **(C)**, and interleukin 10 **(D)**. Effect of LC86 on liver peroxidase activity **(E)**, glutathione level **(F)**, contact enzyme activity **(G)**, and malondialdehyde level **(H)**. Dihydroethidine staining was performed to investigate the effect of LC86 on reactive oxygen species in the liver **(I)**.

Sarcopenia, the age-associated loss of skeletal muscle mass and function, is often accompanied by systemic inflammation. To ascertain the impact of LC86 on inflammatory status, serum levels of pro-inflammatory cytokines (TNF-α, IL-6, and MCP1) and the anti-inflammatory cytokine IL-10 were measured post-intervention in both the LC86-treated and the control (CTL) groups of SAMP8 mice. Compared to the CTL, LC86 administration resulted in a significant reduction in the serum concentrations of TNF-α, IL-6, and MCP1 (*p* = 0.000, *p* = 0.002, and *p* = 0.005, respectively, as shown in Figures 4A–C), while elevating IL-10 levels (*p* = 0.000, Figure 4D). These findings suggest that LC86 exerts a potent anti-inflammatory effect in the context of age-related sarcopenia.

### Enhancement of hepatic antioxidant defense by LC86 in SAMP8 mice

Oxidative stress plays a critical role in the aging process, and the antioxidant defense system is pivotal in mitigating this stress. Our study demonstrates that LC86 supplementation significantly upregulated the hepatic concentrations of superoxide dismutase (SOD) and catalase, alongside an increase in serum glutathione (GSH) and a decrease in malondialdehyde (MDA), a marker of lipid peroxidation (each with *p* = 0.000, Figures 4E–H). Histological analyses corroborated these biochemical findings, revealing reduced reactive oxygen species (ROS) in liver tissues of LC86-treated mice (Figure 4I). Collectively, these results underscore the enhanced antioxidant capacity and improved ROS neutralization conferred by LC86 in the livers of SAMP8 mice.

### Influence of LC86 on gut microbiota diversity and composition in SAMP8 mice

**Figure 5:**
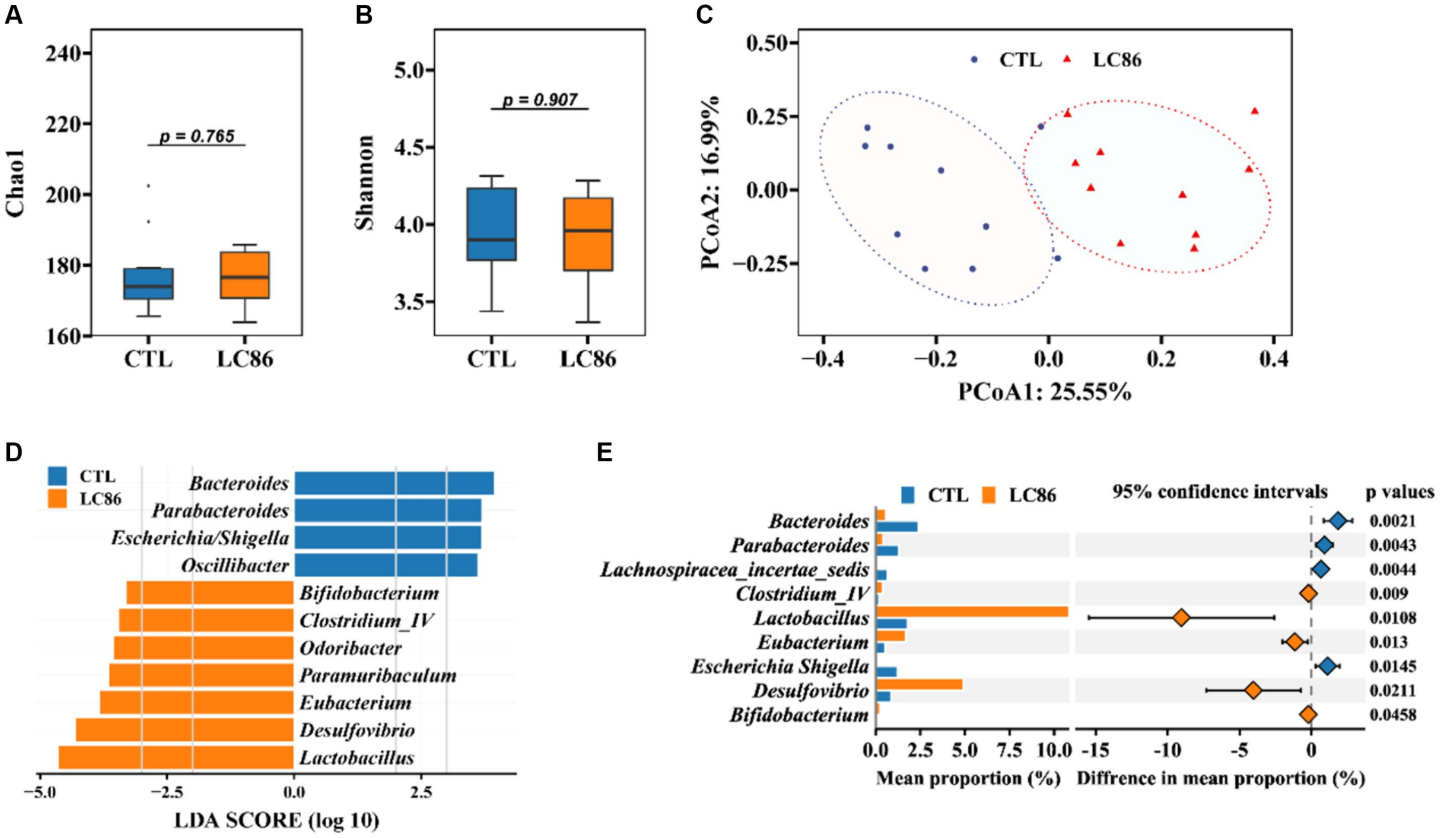
Impact of *Lacticaseibacillus paracasei* LC86 on Gut Microbial Diversity and Composition. The effect of LC86 on the alpha diversity of the gut microbiota was assessed by Chao1 index **(A)** and Shannon index **(B)**, and its beta diversity **(C)**, examined by principal coordinates analysis (PCoA). Additionally, differential species abundance across groups is characterized using Linear discriminant analysis Effect Size (LEfSe) and Statistical Analysis of Metagenomic Profiles (STAMP) **(D,E)**.

The gut microbiota is increasingly recognized for its role in host health and disease, including aging. We assessed the impact of LC86 on the gut microbial ecosystem through alpha and beta diversity analyses. While alpha diversity indices (Chao 1 and Shannon) showed no significant alteration between the groups (*p* = 0.765 and *p* = 0.907, respectively; Figures 5A,B), principal coordinates analysis (PCoA) and permutational multivariate analysis of variance (PERMANOVA) revealed a distinct separation in microbial community structure between LC86-treated and CTL mice (*p* = 0.001, Figure 5C). Furthermore, we utilized LEfSe and STAMP analyses to determine significant differences at the genus level within groups. LEfSe analysis demonstrated that four genera predominated in the CTL group, such as *Escherichia/Shigella* and *Parabacteroides*, whereas seven genera were predominant in LC86-treated mice, including beneficial bacteria such as *Bifidobacterium* and *Lactobacillus* (Figure 5D). STAMP analysis further elucidated the genus-level differences. Compared to the CTL group, LC86 intervention significantly increased the abundance of beneficial genera in the intestinal microbiota of SAMP8 mice, such as *Bifidobacterium*, *Lactobacillus*, and *Eubacterium* (*p* = 0.0458, *p* = 0.0108, and *p* = 0.013, respectively), while markedly reducing the enrichment of harmful genera, such as *Escherichia/Shigella* (*p* = 0.0145, Figure 5E).

### Correlative associations between gut microbiota and host biochemical parameters

**Figure 6:**
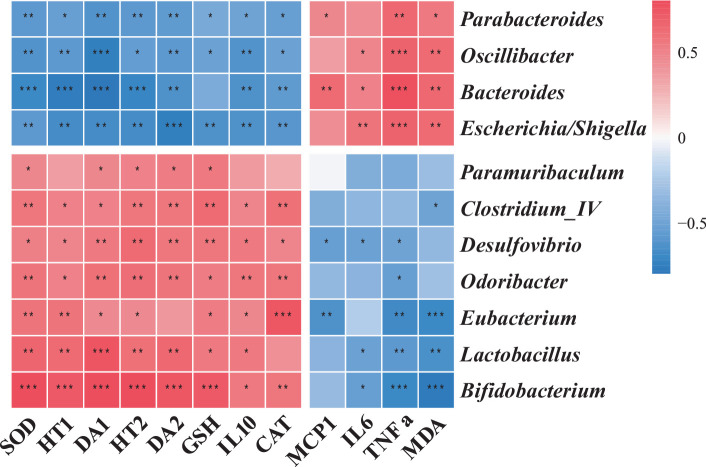
The association of specific microbial species with relevant metadata. TNF α, tumor necrosis factor α; IL6, interleukin 6; IL10, interleukin 10; MCP-1, monocyte chemoattractant protein-1; SOD, superoxide dismutase; CAT, catalase; GSH, glutathione; MDA, malondialdehyde; DA, dopamine, HT, serotonin (5-HT).

Our integrative analysis probed the associations between gut bacterial genera and systemic biochemical markers. Genera enriched in the CTL group, such as *Escherichia/Shigella*, *Bacteroides*, *Parabacteroides*, and *Oscillibacter*, exhibited positive correlations with serum TNF-α and MDA levels. Conversely, genera augmented in the LC86 cohort, including *Bifidobacterium*, *Lactobacillus*, *Eubacterium*, *Odoribacter*, *Desulfovibrio*, *Clostridium*_IV, and *Paramuribaculum*, correlated positively with hepatic antioxidant markers, neurotransmitter concentrations, and IL-10 levels (Figure 6). These correlations highlight potential mechanistic links between gut microbial dynamics and the systemic anti-inflammatory and neuroprotective effects observed with LC86 treatment.

## Discussion

In this study, we explored the therapeutic potential of the probiotic *L. paracasei* LC86 in mitigating age-related sarcopenia and cognitive decline using the SAMP8 mouse model. Our findings delineate a multifaceted improvement in the health span of these mice, characterized by a reduction in the aging phenotype, bolstered muscle glycogen reserves, augmented muscle strength, and enhanced cognitive performance. Furthermore, LC86 administration orchestrated a modulation of neurotransmitter levels in the brain, tempered systemic inflammatory responses, bolstered hepatic antioxidant defenses, and induced a beneficial shift in the gut microbiota composition.

Muscle glycogen, a storage form of sugar in muscle, is broken down for energy when a large amount of blood glucose is consumed during strenuous exercise (38). The augmentation of muscle glycogen content following LC86 treatment, which is positively associated with enhanced muscle strength, echoes the outcomes observed with *L. plantarum* TWK10 supplementation, as documented in prior studies (39, 40). This similarity underscores a potential common pathway via which certain probiotics may bolster muscle energetics and performance (17).

Sarcopenia refers to the age-related loss of muscle mass and function (41), and age-related inflammation is an important etiological factor involved in its development (42–45). The proinflammatory factors TNF-α, IL-6, and MCP1 are reported to be involved in the inflammatory responses leading to the development of sarcopenia (42, 43). IL-10 is a potent anti-inflammatory cytokine that plays an important role in inhibiting the production of proinflammatory cytokines (46). Some studies have speculated that decreased serum IL-10 concentrations may be associated with increased inflammation related to aging (43, 47). In the present study, the LC86 intervention increased the serum IL-10 concentration and decreased serum TNF-α, IL-6, and MCP1 concentrations in SAMP8 mice. Thus, our data suggest that LC86 ameliorates sarcopenia by attenuating the level of inflammation in aged SAMP8 mice. In addition to inflammation, a ROS imbalance has been shown to be associated with brain dysfunction, as indicated by learning and memory decline in older adults (18, 48). We observed that LC86 increased the hepatic antioxidant capacity and reduced the effect of ROS on liver tissue. Therefore, LC86 may have the ability to attenuate ROS and thereby reduce aging-related cognitive impairment and improve memory. Furthermore, the intervention with LC86 positively impacted cognitive and behavioral functions, as evidenced by increased levels of neurotransmitters DA and 5-HT in the striatum and hippocampus of SAMP8 mice. These neurotransmitters are essential for mood regulation, attention, and cognitive processes, and their enhanced levels suggest improved neurotransmitter signaling. Additionally, the increased activity of neuroprotective enzymes such as SOD and glutathione peroxidase highlights the potential of LC86 not only to enhance cognitive functions through neurotransmitter activity but also to provide neuroprotection and slow age-related cognitive decline. This is consistent with prior studies showing that *Lactobacillus* strains like *L. paracasei* LPPS23 and NTU 101 have significant anti-aging, anti-inflammatory, and antioxidative effects, which enhance neurotransmitter levels and stimulate antioxidative enzyme production, underscoring their therapeutic potential for age-related cognitive challenges (49, 50).

**Figure 7:**
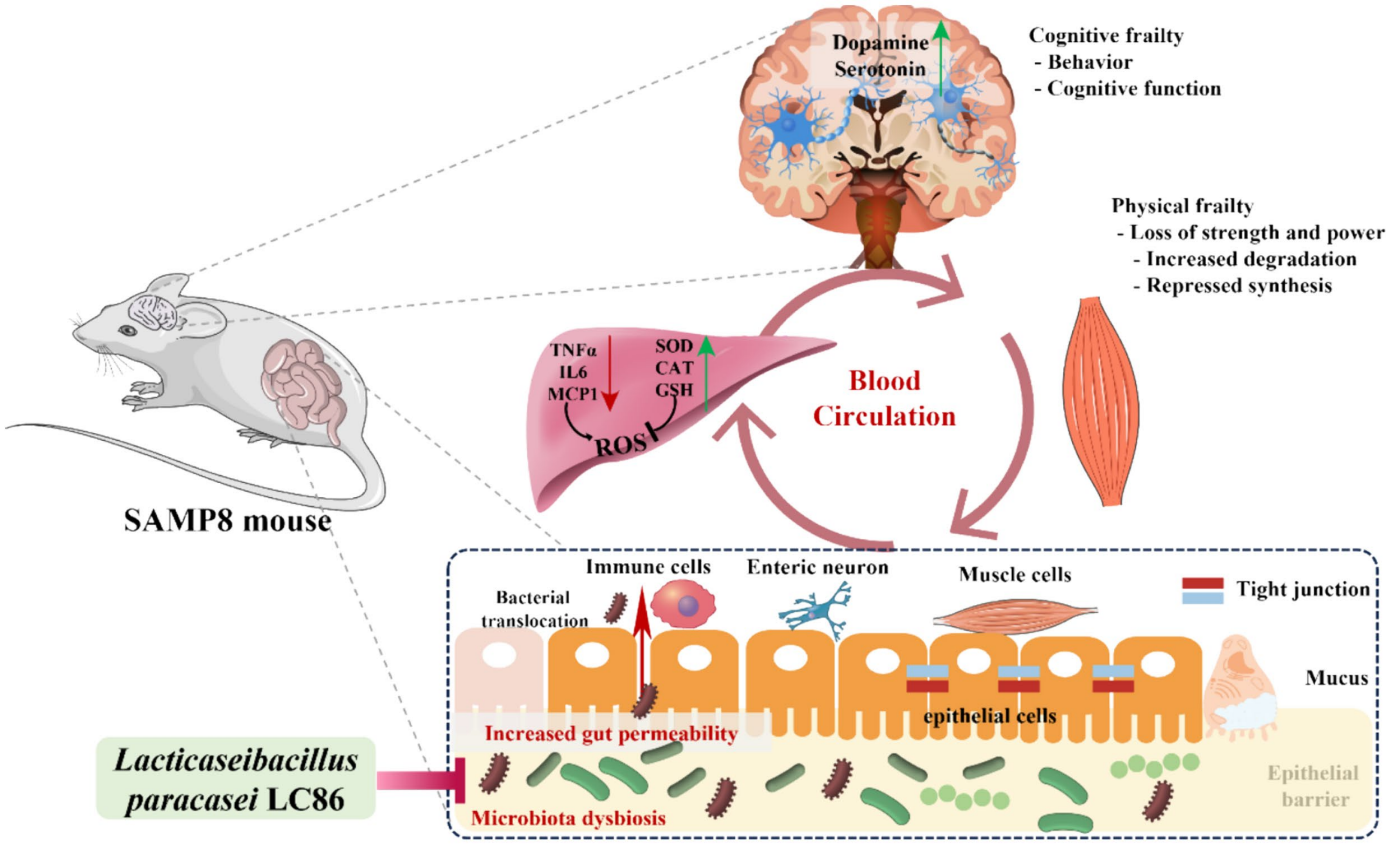
Hypothetical mechanisms by which *Lacticaseibacillus paracasei* LC86 ameliorates age-related muscle wasting and cognitive impairment. TNF α, tumor necrosis factor α; IL6, interleukin 6; MCP-1, monocyte chemoattractant protein-1; SOD, superoxide dismutase; CAT, catalase; GSH, glutathione; ROS, reactive oxygen species.

With increasing age, there is often an imbalance in the gut microbiome (51, 52). An increased relative abundances of some gram-negative bacteria may lead to invasion of the intestinal barrier by endotoxins, resulting in chronic inflammation (53). Research has indicated that probiotic interventions can ameliorate age-associated dysbiosis of the gut microbiota (54). For example, *L. paracasei* J1us66 is capable of enhancing the population of gram-positive bacteria, such as members of the Firmicutes phylum, while inhibiting gram-negative microbes, including those from the phyla Bacteroidetes, Proteobacteria, and Fusobacteria, thus rectifying the structure of the gut microbiome (55). Our study showed that LC86 modulated the gut microbiota, especially by decreasing the relative abundance of *Escherichia*/*Shigella* and increasing the relative abundance of beneficial bacteria, such as *Bifidobacterium* and *Lactobacillus*, in SAMP8 mice. The results of the correlation analysis also showed that LC86 modulated the gut microbiota and that this was associated with improvements in the concentrations of anti-inflammatory factors, neurotransmitters, and hepatic antioxidant-related parameters, all of which had an ameliorative effect on muscle atrophy and cognitive impairment. Based on our experimental results, we hypothesize that LC86 improves the hepatic antioxidant capacity by regulating age-related disturbances in the gut microbiota, which reduces the invasion of harmful bacteria and their products into the liver. The improvement in the gut microbial composition may also regulate brain neurotransmitter concentrations via the brain–gut axis, thereby ameliorating age-related muscle atrophy and cognitive impairment (Figure 7).

Our study provides evidence of the positive effects of LC86 on age-related decline in SAMP8 mice, yet it is not devoid of limitations. The SAMP8 mouse model, selected for its rapid aging characteristics, offers a valuable but not fully equivalent comparison to human aging processes. Therefore, extrapolating these findings to humans should be approached with caution, and further validation through clinical research is necessary. Forthcoming studies might benefit from models that more accurately mirror human physiology or direct clinical trials assessing the impact of probiotics on the elderly. Moreover, our research focused exclusively on the effects of LC86, lacking a comparative analysis with other strains of probiotics. In-depth comparative studies could provide a broader understanding of the differential impacts of various probiotics on the aging process and sarcopenia. Such studies could also include prolonged intervention periods, larger sample sizes, and a variety of aging models to ensure robust findings. This investigation centered on the influence of LC86 on muscle function, inflammatory markers, and gut microbiota, yet it did not encompass all critical aging-related factors. Notably absent were assessments of bone health, cardiovascular function, and reproductive aging, which are crucial aspects of the overall aging process. Finally, the mechanisms underpinning the beneficial actions of LC86 were not delineated. Further research into how LC86 modulates relevant metabolic pathways during aging is essential to deepen our understanding of its role in mitigating aging and reducing the severity of sarcopenia.

## Conclusion

Overall, this study demonstrated that LC86 ameliorated age-related muscle wasting and cognitive impairment in SAMP8 mice through multiple pathways, including increasing the muscle glycogen content, improving muscle strength, regulating neurotransmitter and inflammatory factor concentrations, enhancing the hepatic antioxidant capacity, and regulating the gut microbiota. These results support LC86 as a potential therapeutic agent for age-related sarcopenia and cognitive impairment. Future studies will focus on validating these findings across varied aging models, optimizing supplementation dosages, exploring the impact of probiotic LC86 on human gut microbiota, and confirming long-term safety and efficacy.

## Data availability statement

The datasets presented in this study can be found in online repositories. The names of the repository/repositories and accession number(s) can be found in the article/Supplementary material.

## Ethics statement

The animal study was approved by the Animal Care and Use Committee of Shanghai Laboratory Animal Center and the Ethics Committee of Nanjing Agricultural University. The study was conducted in accordance with the local legislation and institutional requirements.

## Author contributions

YC: Formal analysis, Investigation, Writing- original draft. YD: Methodology, Software, Investigation, Writing-original draft. MH: Writing – original draft. MJ: Writing – original draft. HL: Writing – original draft. ZG: Writing – original draft. KZ: Writing – review & editing.

## Footnotes

^1^https://beikolab.cs.dal.ca/software/STAMP
